# Oral Self-Mutilation in Lesch–Nyhan Patients: A Cross-Sectional Study

**DOI:** 10.3390/jcm11205981

**Published:** 2022-10-11

**Authors:** Gaetano Isola, Ilaria Piccardo, Anna De Mari, Giorgio Alberti, Marco Migliorati

**Affiliations:** 1 Department of General Surgery and Surgical-Medical Specialties, University of Catania, 95124 Catania, Italy; 2 Independent Researcher, 16035 Rapallo, Italy; 3 Orthodontics Department, School of Dentistry, University of Genova, 16100 Largo Rosanna Benzi 10, 16132 Genoa, Italy; 4 School of Dentistry, Genoa University, 16126 Genova, Italy

**Keywords:** Lesch–Nyhan syndrome, disease, clinical study, questionnaire, psychosocial status, oral medicine, dental materials, operative dentistry, oral health, stomatology

## Abstract

Lesch–Nyhan syndrome (LNS) is a rare genetic condition resulting from an inherited disorder of purine metabolism. It is characterized by the lack of one enzyme, hypoxanthine-guanine phos-phoribosyltransferase (HGPRT), which is responsible for purine salvage. The main manifestations of this syndrome are hyperuricaemia, reduction in cognitive abilities, self-aggressive behavior, choreoathetosis, spasticity, and retarded development. The aim of the study was to investigate the means of treatment and efficacy of prevention of oral self-injury behavior (SIB) in patients with LNS. Information regarding the type and treatment of oral SIB in 19 LSN Italian patients (mean age 23.3 years) was gathered via a structured telephone interview of their parents. A total of 84% of the patients showed some form of self-injury behavior; the first form to manifest itself was finger biting (37%), followed by lip biting (25%), and then tongue biting (18%). Furthermore, 74% of cases featured oral SIB, and tooth extraction was found to be the most frequent form of treatment practiced (71%). This study has revealed the great difficulty parents and carers face in managing forms of oral SIB; dental extraction was the most common choice, despite its invasive nature and far-reaching consequences in regard to the psychosocial status of the patients.

## 1. Introduction

Lesch–Nyhan syndrome (LNS) [1] is a rare genetic pathology, whose incidence has been reported to range from 1:1,000,000 to 1:380,000 [2], although the number of known cases would seem to suggest that the incidence is lower [3]. Worldwide distribution of the disease is unknown, but it appears to be uniformly represented in terms of geographical location and ethnic origin. As it involves a defect in the X chromosome, the vast majority of affected patients are males. 

LNS is caused by a genetic dysfunction in purine metabolism and is characterized by a lack of hypoxanthine-guanine phosphoribosyltransferase (HGPRT), an enzyme found in all tissues, especially in the brain, which is responsible for the purine salvage that catalyzes the reaction in which hypoxanthine and guanine are converted to their respective nucleotides [1,4,5].

Clinical manifestations of the disease are hyperuricaemia, reduction in cognitive ability, self-mutilation, choreoathetosis, spasticity, and retarded development [1,2,6,7,8,9,10]. Self-harm, one of the peculiarities of this condition, induces the patients to consciously injure themselves in response to an unwanted but uncontrollable impulse, the product of the disease itself. These patients seek to cause themselves pain in a variety of ways, from the most simple to the most unusual and unpredictable; examples of this self-injury behavior include finger, lip, tongue, and cheek biting; banging the head, arms, or legs against obstacles; inserting fingers or other extremities into dangerous places; or placing their whole person in harm’s way [1,8,11,12].

Patients are aware that their behavior against themselves, and others, is ‘wrong’, but are generally unable to control it; occasionally, they are able to warn their carers of an impending attack, but more often than not they fail, leaving them with a strong sense of disappointment and sorrow. 

Like other forms of self-injury behavior (SIB), oral self-mutilation is seen more frequently in periods of emotional stress, when the patient is uneasy or unwell. The most frequently observed are lesions to the lips, cheek, tongue, and gums, as well as bites to other parts of the body, and self-extraction of teeth. 

Various types of treatment have been proposed for oral self-injury, and can be classed as pharmacological [13,14,15], orthodontic [16,17,18,19,20,21,22,23], or extractive, i.e., the extraction of teeth [24,25]. Pharmaceutical intervention is generally aimed at correcting the dopaminergic deficiency in the striatum, responsible for the self-injury behavior seen in LNS patients. Numerous orthodontic treatments have been suggested, aimed mainly at protecting the areas most affected by oral self-harm or covering the teeth in order to attenuate the effects of mastication during episodes. The most effective solution in these cases is the extraction of the permanent or deciduous teeth, although this can obviously have a great impact of the psychosocial outlook of the patient.

In this context, the objective of this cross-sectional study was to evaluate the incidence of oral SIB in these patients and to assess the frequency of the different treatments used to contrast it.

## 2. Materials and Methods

This was a cross-sectional study on an Italian population of LNS patients contacted through an organization of families affected by the disease (Lesch–Nyhan Group). Data collection was conducted by the Orthodontics and Pediatric Dentistry Department of the University of Genoa. The period of recruitment was between September 2017 and June 2018. The parents of 19 patients were contacted and asked to complete a structured interview reported in Table 1. The primary objective was to evaluate incidences of SIB and secondarily report the treatment options to manage this condition. The questions asked investigated the current and past clinical histories, the odontostomatological situation, and the occurrence of SIB. At the moment of interview, two of the patients were already deceased but were included in the study thanks to the information kindly provided by their parents.

## 3. Results

The age of the patients ranged from 5 to 46 years, with a mean age of roughly 23.3 years (±9.4); diagnosis of the condition had been made at a mean age of 4.7 years (± 3.0).

### 3.1. Self-Injury Behavior 

**Figure 1:**
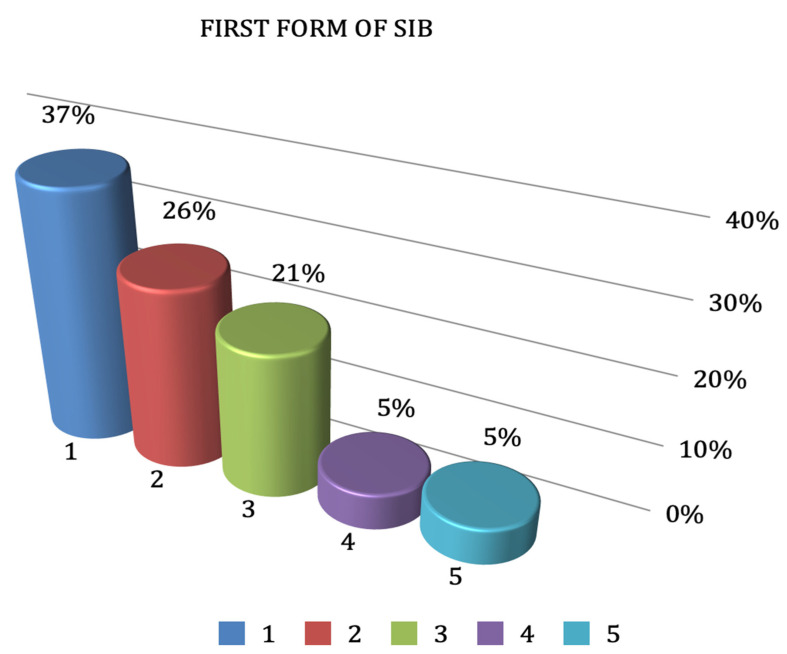
Incidence of the first forms of self-mutilation. 1: finger biting; 2: lip biting; 3: tongue biting; 4: throwing oneself backwards; 5: head banging.

According to the parents, 16 patients out of the 19 included in the sample displayed some form of self-injury behavior (84%). The first form of self-mutilation to manifest itself was found to be finger biting 7/19 (37%), followed by lip biting 5/19 (26%) and tongue biting 4/19 (21%). A total of 14 patients out of these 16 displayed oral SIB (74%) (Figure 1).

Among patients displaying oral self-mutilation, the most frequent was lip biting 17/19 (89%), followed by tongue biting 11/19 (58%) and cheek biting 10/19 (53%). Less frequent manifestations were vestibular and gum lesions, each seen with a frequency of 1/19 (5%). Furthermore, 12 of the 14 patients manifested permanent lesions, six of which were to the lower lip.

The three patients (16%) showing no signs of self-mutilation were the youngest, respectively 5, 8, and 9 years of age, the age range during which this kind of behavior generally begins to manifest itself.

A total of 42% (8/19) of all patients included in the sample displayed grinding behavior. Moreover, 71% (10/14) of these had consequently been treated by means of extraction, while orthodontic appliances were employed in 29% of cases (4/14).

### 3.2. Treatment for Self-Injury

**Figure 2:**
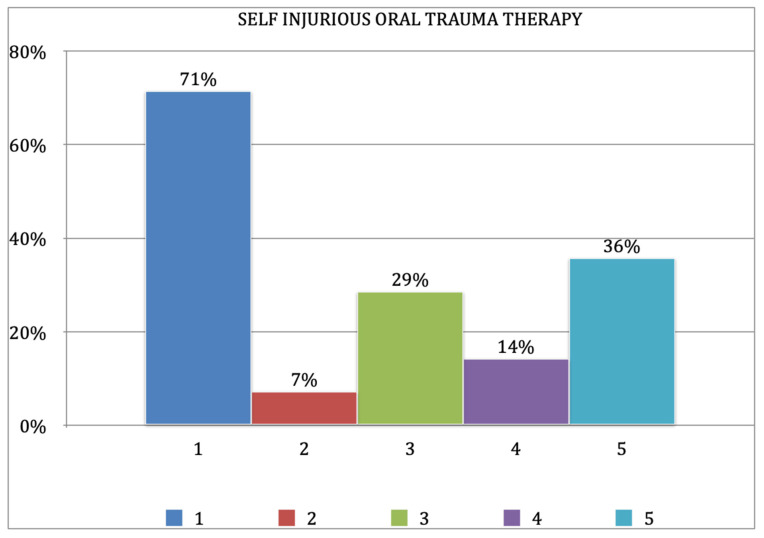
Incidence of the various forms of therapeutic approach. 1: tooth extraction; 2: drugs; 3: orthodontic appliances; 4: dental re-contouring; 5: obstructive methods.

The majority of patients had been subjected to extraction (71%); of the ten patients with extracted teeth in our sample, extraction had only been performed for apparently therapeutic reasons in six cases; two other patients had self-extracted, one had lost their teeth due to the lack of dental treatment, and another due to combined trauma and lack of treatment. The remainder of the patients due to orthodontic appliances as, for example, an acrylic maxillary device, designed and constructed with an occlusal plate raising the bite or soft resin mouth guard (29%). Other types of treatment had been used with limited success, including filing the teeth to make them less likely to cut the oral and perioral tissues (14%); painkilling drugs (7%) for palliative rather than symptomatic relief; and obstructive methods such as placing objects between the teeth to prevent biting lesions (36%) (Figure 2).

## 4. Discussion

The syndrome was diagnosed at a mean age of 4.7 years (±3.0), often upon the birth of a second child affected by the same syndrome. Only five patients were diagnosed in their first year of life, eight patients got their diagnosis between one and six years of age, and the remainder were provided with a precise diagnosis after seven years of age. 

Three pairs of siblings suffering from the same condition were among the 19 patients recruited. This appears to be a fairly frequent occurrence due to the lack of a precise diagnosis in the firstborn; indeed, it is often only the birth of the second afflicted child that reveals the existence of the pathology. 

Considering lifetime self-injury behavior, the above-mentioned results are similar to those published by Anderson et al. who reported a 90% permanent physical damage with compulsive approach [26].

The first form of self-injury to manifest itself in the group was finger biting (37%), followed by lip biting (26%), and finally tongue biting (21%). Other, less frequent forms of self-harm were general biting and throwing oneself backwards, as well as banging the head (5% for each).

These kind for lesions are those typically observed in LN patients and reported in other studies [27].

Of these 16 self-harming patients, 14 (74%) displayed oral self-injury behavior. However, the two non-oral self-mutilating patients did display tooth grinding behavior in times of great emotional stress, during fever, or when ill. In fact, it is worthy to note that the majority of patients displayed, or have displayed, more than one form of self-injury behavior at the same time, or had developed various forms over the years. 

Accordingly to Anderson, who reported permanent damages over 45% of those patients, in the present study the majority of patients (86%) also showed permanent lesions following oral self-injury behavior. The most prevalent of these were of the upper lip; six patients (50%) had bitten their lower lip completely off, while the others displayed at least one scar.

Biting scars were also found inside the cheeks, on the buccal mucosa, in seven patients, and, less frequently, presumably due to its great regenerative capacity, on the tongue. This notwithstanding, two patients had completely bitten off a portion of their tongue as parents reported; one, the tip using the incisors; and the other two lateral portions—the anterior using the canine and the posterior using the molars. Two patients also had resection scars on the fingers and missing fingernails, and one patient had a scar accompanied by missing tissue at the left nostril. 

Bruxism and tooth grinding were very common in these patients. As previously mentioned, two patients who did not display other forms of self-injury ground their teeth at night, and at times of emotional stress. In addition to these two patients, grinding was reported in five other patients who displayed oral SIB, and in one who displayed a non-oral pattern of self-harm. Thus, 8 out of the 19 LNS patients considered presented grinding (42%).

Various solutions have been proposed to prevent oral self-injury. These involve positioning orthodontic or other devices between the teeth, pharmacological therapy, and tooth extraction. However, the management of these cases is difficult [28,29], and parents must be involved in the clinician’s decision-making process [30].

Some of the prevalent non-orthodontic means of preventing biting injuries were reported to be dummies (pacifiers); a roll of gauze, buccal shields, or other cloth or rubber objects placed between the teeth; and a plaster to stick the lower lip to the chin, thereby distancing it from the teeth.

The literature contains a variety of case reports proposing the use of various appliances for limiting oral SIB, as soft mouth guard fabricated to prevent the destruction of perioral soft tissues and combined psychiatric pharmacologic therapy have been proved to have satisfactory results [16,17,18,19,20,21,22,23]. On the whole, these have been successful at protecting the oral tissues and other parts of the body. Extraction of the teeth, on the other hand, is the most invasive of the treatment options available and leads to significant oral disability even though it led to improvement of patients’ lesions. Despite this, it was the most commonly observed solution in our study, probably due to the possibility to an immediate solution of the patient’s problem. Indeed, the telephone interviews conducted in this study revealed that 6 out of 19 patients were not regularly (once a year) examined by a dentist (31%). Of the remaining 13 carers interviewed, 5 would not or could not respond to this question, suggesting that this percentage may, in fact, be far higher.

Interestingly, two pairs of parents who had consented to this procedure being performed on their children, despite having been informed about the tragic consequences, now regret their choice. 

In our sample, the extractions had been performed at different ages; several had been carried out in childhood, with the entire set of deciduous teeth being extracted, while the majority had been performed in permanent dentition, either extracting all the teeth in one sitting under general anesthesia, or extracting the teeth, or parts of teeth, progressively on the basis of the lesions provoked.

Following the extraction of all his deciduous teeth, one patient stopped biting himself and showed no further oral problems, thereby obviating the need for extraction of the permanent teeth; this could be an interesting approach to be investigated further to assure that this approach can effectively reduce or delete SIB in permanent dentition.

## 5. Conclusions

- Self-injury behavior in Lesch–Nyhan patients represents a severe management problem that both parents and oral health carers have to face. The incidence of SIB reported is 74% and led to permanent damages in 86% of cases. Among different therapeutical options, including oral appliances and drug administration, dental extraction is the most frequently chosen therapy (71%) despite its invasive approach;
- This cross-sectional study shows how extraction therapy is extremely distressing for patients and suggests that the use of orthodontic appliances reported in the literature should be preferred wherever possible in order to improve the patients’ quality of life.

## Figures and Tables

**Table 1:** Questionnaire used for the study.

Year of birth
Age at Lesch–Nyhan Syndrome diagnosis
Presence/absence of self-injury
First form of self-injury
Presence/absence of oral SIB
Type of oral SIB
Presence/absence of permanent oral lesions
Type of permanent lesions
Treatment for oral SIB
Extractions
Age at which extractions were performed
Drugs
Orthodontic appliances
Dental check-ups

## Data Availability

Data are available from the corresponding author upon reasonable request.
